# Computer-Aided Detection and Diagnosis in Medical Imaging

**DOI:** 10.1155/2013/790608

**Published:** 2013-09-01

**Authors:** Chung-Ming Chen, Yi-Hong Chou, Norio Tagawa, Younghae Do

**Affiliations:** ^1^Institute of Biomedical Engineering, National Taiwan University, Taipei, Taiwan; ^2^Department of Radiology, Taipei Veterans General Hospital and National Yang Ming University, Taipei, Taiwan; ^3^Division of Information and Communication Systems, Tokyo Metropolitan University, Tokyo, Japan; ^4^Department of Mathematics, Kyungpook National University, Kyungpook, Republic of Korea

Medical images nowadays play an essential role in detection and diagnosis of numerous diseases. Ranging from anatomical information, functional activities, to the molecular and cellular expressions, medical imaging provides direct visualization means to see through the human bodies and observe the minute anatomical changes and biological processes characterized by different physical and biological parameters. Informative as they are, medical imaging usually requires experienced medical doctors to best interpret the information revealed in the images. However, because of various subjective factors as well as limited analysis time and tools, it is quite common that different medical doctors may come up with diverse interpretations, leading to different diagnoses. Moreover, for the same set of medical imaging, a medical doctor may make different diagnosis results at different time. 

To attain a more reliable and accurate diagnosis, recently, varieties of computer-aided detection (CAD) and diagnosis (CADx) approaches have been developed to assist interpretation of the medical images. At least four types, denoted as Types I–IV, of efforts may be identified among these CAD and CADx approaches. Type I is to assist visual detection, qualitative analysis, and interactive quantitative analysis of the objects of interest in the medical images by either enhancing the salient features of the objects or suppressing the background noises. Type II is to assist feature extraction of the objects of interest for further quantitative analyses by such techniques as boundary delineation, tree-structure reconstruction, fiber tracking, texture analysis, and so on. Type III is to automatically detect and classify the objects of interest by integrating the data mining, medical image analysis, and signal processing technologies. Type IV is to estimate the anatomical and functional tissue properties not explicitly revealed in the medical images based on mathematical modeling, for example, physiology, biomechanics, heat transfer, and so forth.

This special issue presents one review paper and fifteen papers of latest research results on computer-aided detection and diagnosis in medical imaging covering all four types of works, including one paper of Type I, six papers of Type II, seven papers of Type III, and two papers of Type IV. The distribution of these four types of research works, though only with a limited number of papers, does reasonably account for the amount of research efforts in the various areas of CAD/CADx in medical imaging. 

The Type I paper in this special issue, presented by Y. Dai et el., proposed a volume-rendering-based interactive 3D measurement framework for quantitative analysis of 3D medical images. The idea is to integrate 3D widgets and volume clipping into volume rendering, using 3D plane widgets, 3D line widgets, and 3D angle widgets to measure the areas, distances, and angles of interesting objects.

To assist feature extraction and quantitative analysis, the six papers of Type II in this special issue may be further divided into three groups, namely, registration, texture analysis, and segmentation, representing three essential tasks in CAD/CADx in medical imaging. The registration group has only one paper in which R. Xu et al. addressed one of the key issues in medical image registration, that is, determination of corresponding points. A particle-system-based method was proposed to obtain adaptive sampling positions on the unit sphere for the construction of statistical shape models. In the group of texture analysis, D. Avola et al. presented a customizing approach to deriving a set of first- and second-order statistics-based operators for texture analysis of MRI images. J. Deng et al. proposed a robust statistical texture model for medical volumes based on a linear tensor coding (LTC) algorithm. Medical volumes are represented by a linear combination of mutually independent bases, from which distinctive bases may be selected for classification. To extract the morphological information, in the segmentation group, Y. Ujihara et al. developed a two-step region growing method to reconstruct cell geometry from confocal fluorescence microscopy images of the cytoskeleton. C.-F. Jiang et al. integrated the GVF-snake model and a hybrid registration technique to extract regions from MR T1-weighted images, mapping them into the corresponding SPECT images. To automatically recognize the vertebral column in a SPECT image, S.-F. Huang et al. formulated the bone segmentation problem as a graph clustering problem and proposed a “bone graph” image description method to facilitate manipulation of morphological relationships in the skeleton. 

To assist nodule detection and/or differential diagnosis of various diseases, the seven papers of Type III in this special issue presented CAD/CADx approaches for six clinical applications. They are mammographic lesion detection and diagnosis, lung nodule detection, as well as differential diagnosis of Alzheimer disease (AD) versus mild cognitive impairment (MCI), cerebral lymphomas versus glioblastomas, pancreatic diseases, and trigger finger disease. As a review paper, T. Ayer et al. provided an informative overview of artificial neural networks-based mammography interpretation and diagnostic decision making. Both of B. Li et al. and A. Tartar et al. aimed to achieve computer-aided detection of lung nodules in CT images. Nevertheless, A. Tartar et al. focused on classification of a candidate nodule into true nodule or nonnodule by selecting the best features from three conventional methods. On the other hand, B. Li at al. proposed a complete framework for nodule detection based on a fuzzy integrated active contour model and a hybrid parametric mixture model of the juxtavascular nodules. For differential diagnosis, S.-T. Yang et al. proposed an MRI-based classification framework to distinguish the patients with AD and MCI from the normal participants, using particle swarm optimization for feature selection and support vector machine as the classifier. To differentiate cerebral lymphomas from glioblastomas, T. Yamasaki et al. presented a tumor classification system, classifying typical cases by luminance range thresholding and apparent diffusion coefficients thresholding and nontypical by a support vector machine (SVM). A. Jiang et al. developed a classification method for pancreatic diseases, using multilinear principal component analysis to extract the eigen tensors and SVM as the classifier with the parameter optimized by a quantum simulated annealing algorithm. Y.-C. Liu et al. proposed two parameters as the pathological progression indices for evaluation of trigger finger disease from the microscopic pulley images. These two parameters are the size ratio of the abnormal tissue regions and the number ratio of the abnormal nuclei, derived from a color-based image segmentation system.

Both of the two Type IV papers in this special issue aimed to estimate the anatomical and functional tissue properties from MRI images. With dynamic MR images, Y.-H. Kao et al. investigated the respiratory and cardiac pulsations in the brain of normal subjects based on transfer function analysis. W. Swastika et al. analyzed two statistical models of diaphragm motion constructed by using regular principal component analysis (PCA) and generalized N-dimensional PCA (GND-PCA), the results of which showed that the GND-PCA model was superior to the PCA model.

*Chung-Ming Chen* *Yi-Hong Chou* *Norio Tagawa* *Younghae Do*

